# The Teaching Strategy of Socio-Political Education by Deep Learning Under Educational Psychology

**DOI:** 10.3389/fpsyg.2022.910677

**Published:** 2023-02-07

**Authors:** Zhen Chen, Lan Wen, Xiaoqing He, Peiyao Chen, Hua Wu

**Affiliations:** ^1^School of Marxism Northeastern University Shenyang, Shenyang, China; ^2^South China Business College, Guangdong University of Foreign Studies/Faculty of Education, Guangxi Normal University, Guangzhou, China; ^3^School of Marxism, Chengdu Normal University, Chengdu, China; ^4^School of History and Culture, University of Birmingham, Birmingham, United Kingdom

**Keywords:** educational psychology, deep learning, socio-political education, teaching strategy, teaching strategies

## Abstract

This study aims to optimize the teaching content of ideological and political courses and guide students to establish correct values. Inspired by Artificial Intelligence, the K-means clustering algorithm was applied to the neural collaborative filtering algorithm through temporal data. Besides, a deep learning algorithm was designed for the improved matrix factorization. The evaluation indicators were selected through experiments. The relevant data sets were used for simulation and testing. The test results indicated that the Root Mean Square Error of this scheme was 1.251, and the Mean Absolute Error was 0.625. These index measurement values were better than those of similar algorithms, indicating this model has better performance after optimization and can recommend suitable courses. The innovative algorithm designed to construct the classroom teaching model of social and political education can accurately recommend proper courses according to the students’ learning situation reflecting their psychological states. The research provides adaptive teaching for students, enables interaction between teachers and students, and helps students form correct values. It also has an important role in improving the teaching strategies.

## Introduction

Students are a vital force for the future development of a country, who form their outlook on life during the campus period (Qian et al., 2018). With a proliferation of information, students are easily adversely affected by complex information that can distort their perspectives. Therefore, all parties with educational responsibilities must strengthen education and guidance of students’ outlook on life to ensure the quality of talents (Chen, 2019). At present, contemporary educational psychology is increasingly frequently combined with information technologies for cross-disciplinary research, such as deep learning (DL) technology (Rogoza et al., 2018). The recommendation algorithm based on DL is common in the education field. The performance of the recommendation algorithm has a key impact on using educational psychology theory to provide students with adaptive teaching, so it is crucial to improve the performance of the recommendation algorithm (Wu et al., 2019).

The development of recommendation algorithms mirrors the development of recommendation systems (Fang and Lu, 2021). The earliest recommendation algorithms were designed to bring personalized movie viewing services to movie watchers. Later, some scholars applied collaborative algorithms to recommender systems, such as the item-based collaborative filtering algorithm (CFA). With the continuous development of recommender systems, there are an increasing types of recommendation methods, such as content recommendation, collaborative filtering, and hybrid intelligent recommendation (Wu et al., 2019). They offer many possibilities for modern education and personalized recommendation education. However, when data values are missing, these recommendation algorithms face the problem of accuracy decline and data sparsity (Wu et al., 2020).

The research motivation is to resolve the problem of data sparseness by using an improved recommendation algorithm based on the neural CFA combining the advantages of DL and traditional CFA (Wu and Song, 2019). Traditional Matrix Factorization (MF) was used to discover the linear relationships between users and items. Besides, multi-layer neural networks were used to explore the nonlinear relationships between users and items to reduces the interference of missing data values and optimize the data accuracy. Curriculum resources should change as teaching continues to improve. The K-means clustering algorithm was applied to neural CFA through temporal data to maximize course resource optimization. The relevant evaluation indicators were selected through experiments on relevant datasets. The experimental results demonstrated that the optimized algorithm showed good performance. The novelty of the research lies in using neural CFA to design an algorithm model to recommend suitable social and political courses according to the actual situation and promote teacher-student interaction. The research findings make an essential contribution to improving teaching strategies in ideological and political courses.

## Recent Related Work

Ren et al. (2021) predicted the high dropout rate faced by the MOOC platform by combining DL and ensemble learning. The authors used convolutional neural networks to extract hidden features from raw data and utilized the output features as the input of the ensemble learning model. Then, various traditional classification methods are used for training and prediction, and the prediction results of various models are fused as the final result. The research has important reference value for constructing the prediction model of students’ dropout behavior. Troussas et al. (2020a) proposed a framework employing artificial neural networks and weighted models for students’ collaborative learning styles to provide learners with recommendations for collaborative activities. The authors evaluated this framework through statistical hypothesis testing. The results showed that the proposed method was highly pedagogical and practical and positively impacted learning outcomes. Pu et al. (2020) put forward a general framework for AI planning or decision-making for teaching sequencing of deep reinforcement learning frameworks. Both simulations and experiments in the classroom confirmed the validity of the research method. Troussas et al. (2020b) programmed computers with a teaching strategy of adaptive learning activities. The results showed that the proposed method outperformed other methods that lacked adaptability in terms of domain knowledge and learning theory, significantly improving student learning ability. Citakoglu (2021) conducted long-term monthly temperature estimation and research in Turkey by comparing multiple Artificial Intelligence learning models (Li et al., 2019). They proved the validity of the proposed method through a survey of monthly air temperature using data from 250 measuring stations in Turkey. To sum up, the research on adaptive teaching strategies in psychology from the perspective of educational psychology has important reference value for applying DL in the education field. This work studied the improved recommendation algorithm, and the test results demonstrated the excellent performance of the optimized algorithm. It can provide appropriate course recommendations on social and political courses and facilitate the improvement of course resources and teaching methods.

## Materials and Methods

### Research on Educational Psychology and Teaching Strategies

The socio-political courses at schools are an important part of students’ outlook on life education to help them develop a healthy and positive attitude and establish a correct outlook on life. However, there are still many problems in the students’ view of life education. Educators need to identify these issues and use appropriate methods to address them (Yuan and Wu, 2020). It is the rigid requirements for the development of students’ view of life education to conform to the times and apply new educational concepts (Wu et al., 2021). Only by following the laws of students’ psychological development and educational principles can education guide students to establish a correct outlook on life in a good and positive way and promote their healthy and positive growth (Wu and Wu, 2017). Educational psychology is a process of studying the basic laws of teaching and learning in the educational and teaching environment, analyzing and optimizing teaching and learning methods, and exploring students’ abilities and potentials. In short, educational psychology is a psychology-based discipline to study humans’ learning, educational intervention, instructional psychology, and social psychology in the context of education (Zheng et al., 2018). Educational psychology focuses on the application of psychological theory or the results of psychological research to education, including designing curriculum, to promote students’ academic motivation and help students face setbacks in life, learning, and growth.

**Figure 1:**
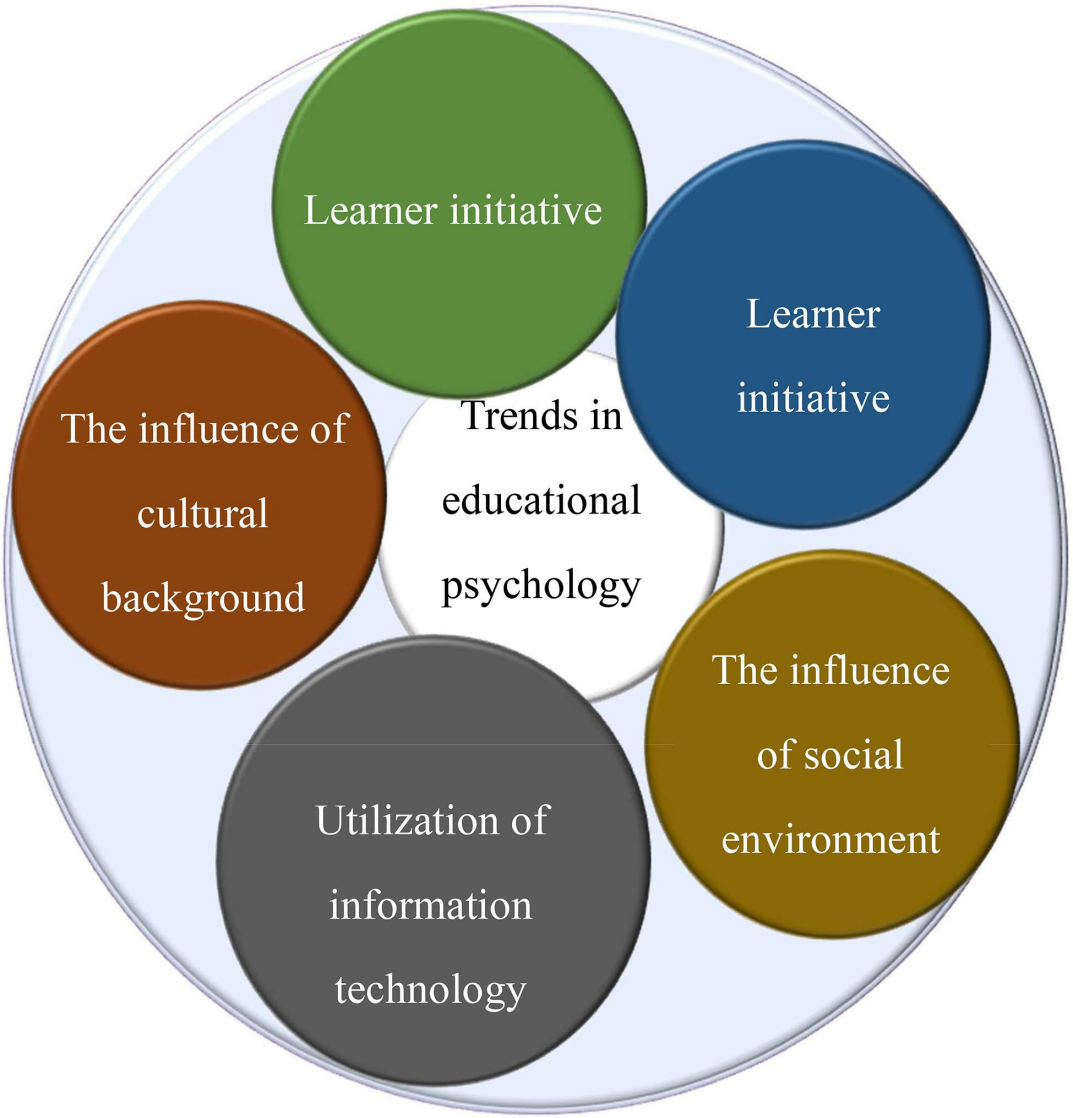
Trends in educational psychology research.

Educational psychology primarily studies students’ mastery of knowledge and skills and the psychological phenomena and development laws under the conditions of education and teaching, such as their moral standards and personality formation. Therefore, educational psychology has unique characteristics different from pedagogy and psychology. Learning and teaching is an interactive system running through the entire learning process, involving factors such as students, teachers, teaching content, teaching environment, and teaching media. The teaching process and the evaluation process interact and have the nature of pedagogical and psychological tasks. The essential features, functions, and meanings of educational psychology can be summarized through retrospective, quantitative, and qualitative methods, which is of great significance for promoting the development of educational psychology. With the development of society, the application of information technology becomes the research content of educational psychology. Figure 1 presents research trends in educational psychology (Xiao and Shen, 2019).

As shown in Figure 1, the research trend of educational psychology lies in applying information technology to fully mobilize the learner’s autonomy and initiative and improve learning interest and learning Effect under the influence of the social environment and cultural background. Therefore, DL technology was used in educational psychology to analyze the curriculum content that students need and help teachers analyze their learning situation in this paper.

### Course Recommendation Algorithm

**Figure 2:**
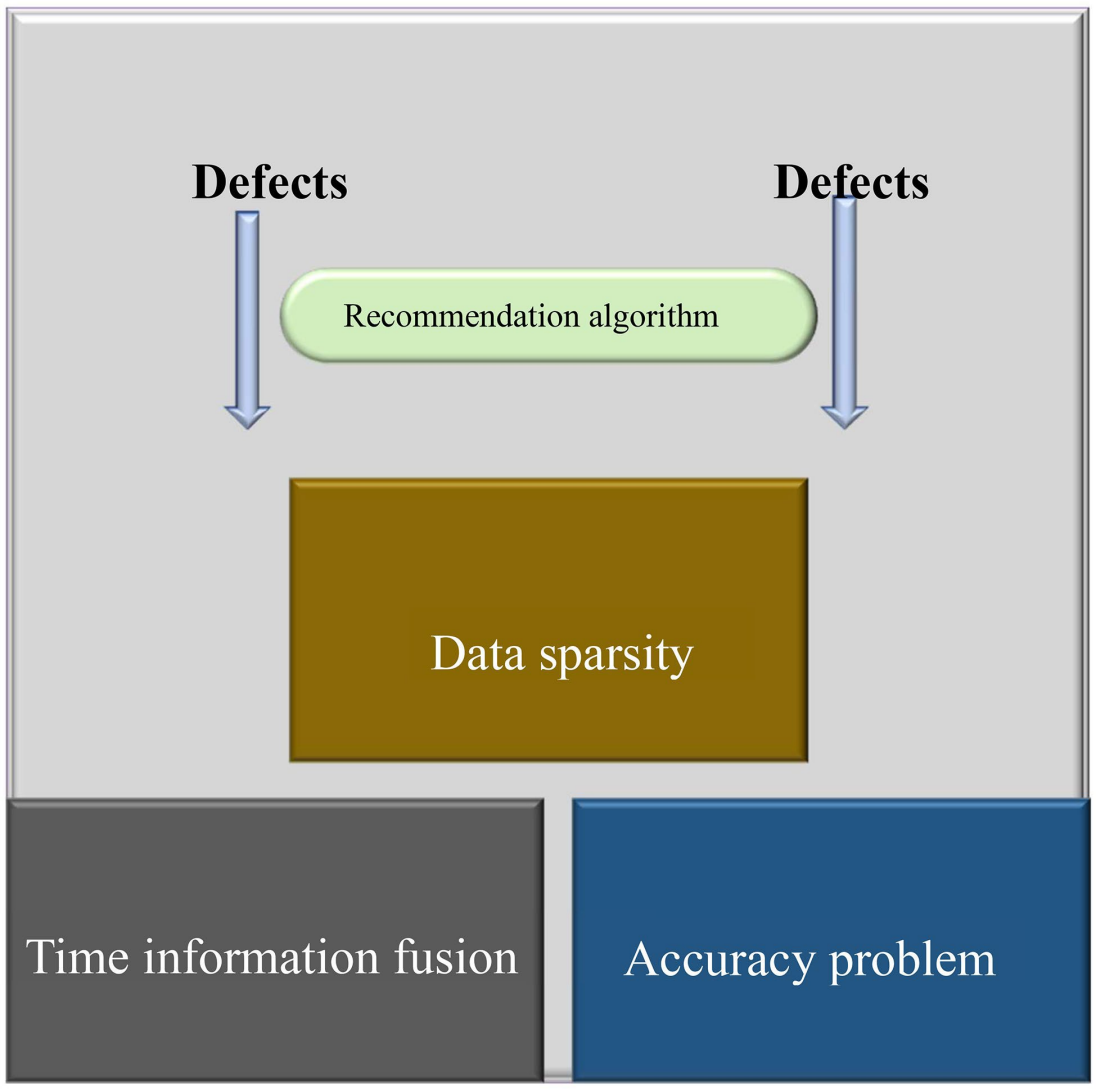
Deficiencies of the existing course recommendation algorithm.

At present, the newly developed course recommendation algorithms have the some problems need to be solved, as shown in Figure 2.

The sparse data in the course recommendation algorithm presented in Figure 2 is because the previous CFA offers relevant recommendations based on the analysis of the user’s past information (Xiao et al., 2018). However, information on user ratings of relevant items is incomplete, and it is impossible to determine whether the ratings are accurate in actual use environments. Scoring may become a redundant item after a user’s coursework is complete. Correspondingly, users do not perform scoring operations, resulting in fewer data sources (Zhu et al., 2018). The accuracy of recommendation algorithms cannot be guaranteed under the combined effect of two factor: the continuous update and development of learning resources and the dynamic learning process of users (Suganya et al., 2020). Furthermore, temporal information is quite different from auxiliary information. Temporal information has no intervals but is continuously increased due to the influx of other data; besides, temporal information is not directly input (Perrotta and Selwyn, 2020).

Neural networks can discover high-level features while learning nonlinear networks of users and objects, resolving the above three problems and ameliorating data loss.

### Neural Networks in Deep Learning

Teachers can use the course recommendation system to teach students the actually necessary knowledge (You, 2019). Due to the lack of face-to-face communication between teachers and students in the process of remote teaching, teachers just consider the learning situation of most students in the process of explaining knowledge. This condition may lead to a gradual disconnect between the classroom lessons and students’ needs (Knowles, 2018). Course recommendation systems can efficiently solve this problem. The combination of DL and traditional recommendation algorithms can help teachers gain a comprehensive understanding of students’ individual needs (Li and Ye, 2020).

**Figure 3:**
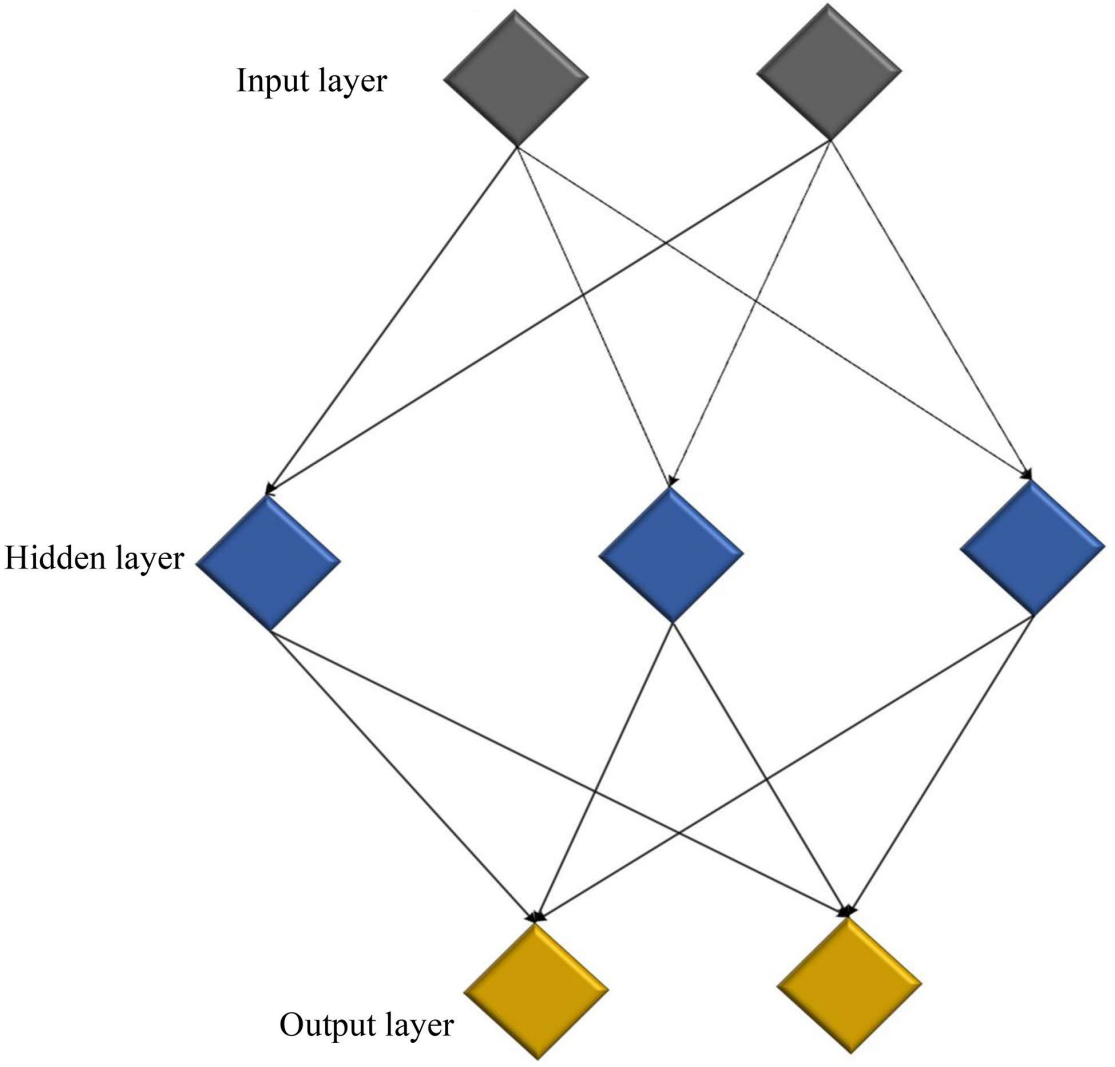
Structure of the neural network.

Neural networks can input complex problems into algorithmic models for analysis. According to the characteristics of the problem, the relationship between the autocorrelation nodes in the model is adjusted to improve the processing efficiency. A neuron is the most fundamental unit of a model. Each neuron can receive multiple inputs. The weight of the input data affects the entire neuron (Fan et al., 2021). Figure 3 displays the neural network composed of neurons.

In a neural network, the function of the input layer is to receive external signals. The hidden layer is usually located between the input layer and the output layer. The network finds suitable solutions through data processing and analysis and continuous network learning and training (Wang, 2021). A neural network’s structure determines its output.

In a neural network, input data is transformed into output data at the nodes of each layer and then applied to the next node (Nassar et al., 2020). The activation function refers to the functional relationship between two factors to efficiently transform the output of the previous layer into the input of the next layer. It can also map from a neuron’s input to its output. Using an activation function can turn the linear relationship between the input and output of a network layer into a nonlinear one to explore deep relationships. The most frequently used activation functions are the Sigmoid function, Tanh function, and Relu function (Bobadilla et al., 2020).

The input values and output results of the Sigmoid function all range between 0 and 1. It can achieve satisfying results when the differences in object characteristics are not particularly obvious. The problem of vanishing gradient often exists due to the relatively large amount of calculation of the function. The conversion of the input x through function *S* can be expressed as Equations (1) and (2).


(1) $$S\left(x\right)=\frac{1}{1+e^{-x}}$$

(2) $$S^{\prime }=\frac{e^{-x}}{{\left(1+e^{-x}\right)}^{2}}=S\left(x\right)\left(1-S\left(x\right)\right)$$

The Tanh function is a hyperbolic function, which can make up for the asymmetry of the Sigmoid function with the origin. Still, it has the disadvantage of gradient saturation. The Tanh function can be written as Equation (3).


(3) $$Tanh\left(x\right)=\frac{e^{x}-e^{-x}}{e^{x}+e^{-x}}$$

The results of the ReLU function do not suffer from gradient saturation or descent compared with the Sigmoid function and Tanh function. In practical applications, this function is only used to find the maximum value not to calculate the exponent. However, when the function learns or is trained, there are some other problems with gradients, such as neuron deactivation (Chen et al., 2019). Equations (4) and (5) indicate the relationship between the function ReLU and the input x.


(4) f(x)=max(0,x)

(5) ReLU(x)={x,ifx>00,ifx≤0

### CFA Model *via* Neural Networks

**Figure 4:**
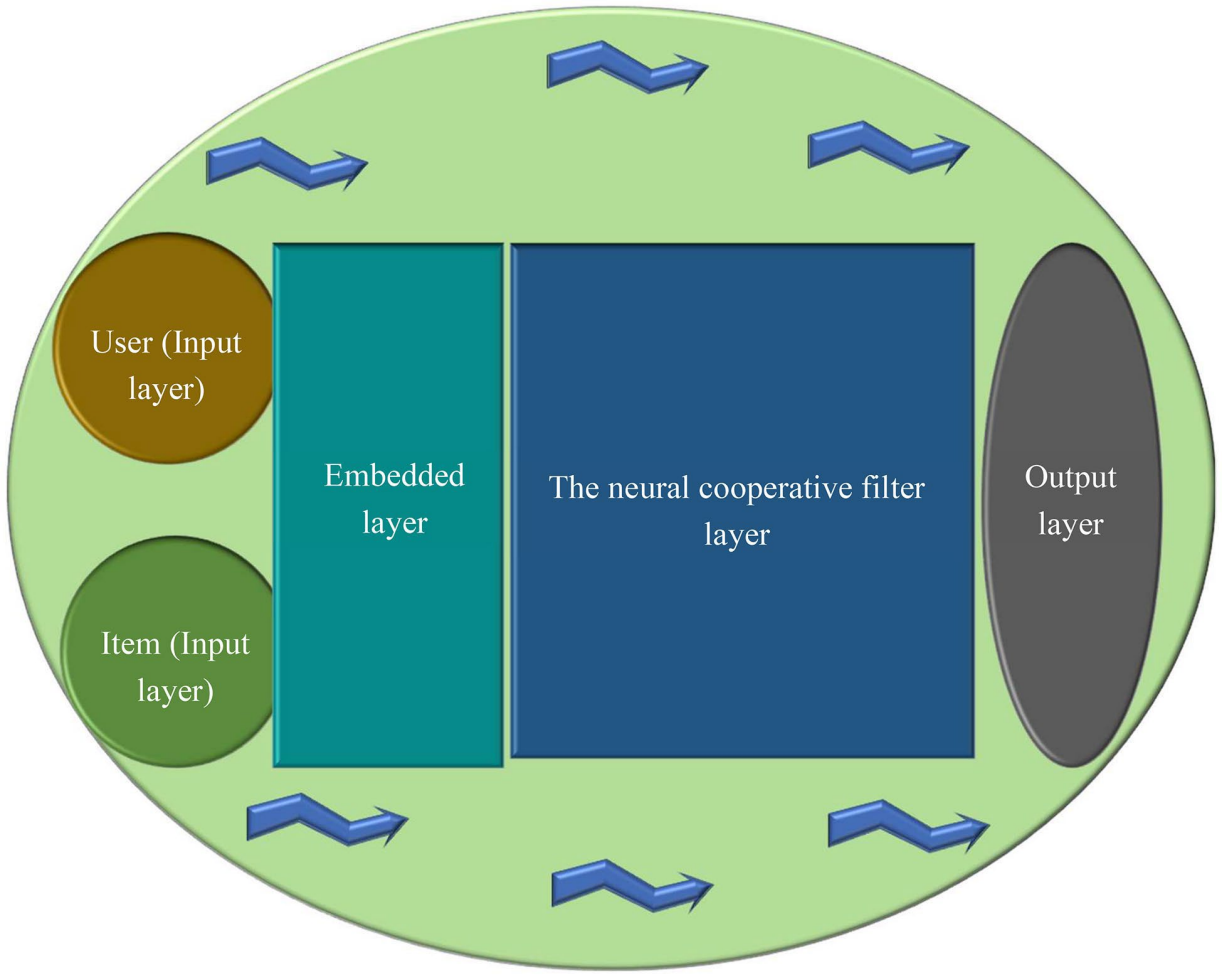
Framework of neural collaborative filtering algorithm (CFA).

CFA can achieve excellent recommendation effect, so it is frequently used. Its working principle is to classify users according to their preference on the basis of analyzing their historical behavior data and then recommends similar products for users of the same category. CFA is also a research hotspot, and researchers have optimized the CFA for recommendation to improve the performance (Fu et al., 2018). Existing CFAs use MF to construct hidden features for users and items. However, a simple inner product cannot accurately estimate the complex relationship between users and items, limiting the recommendation accuracy. This study used a neural CFA model to replace the inner product operation in the standard MF to discover the linear and nonlinear relationship between users and items. Figure 4 illustrates the specific framework of the neural CFA.

The neural CFA model in Figure 4 consists of four layers: the input layer, embedded layer, user layer, and output layer. The input layer takes input users and items and converts them into vectors. For example, the input of 𝑛 users needs to be converted into a vector of 1 × 𝑛, which can be converted into a sparse vector. After the input reaches the embedded layer, the input vector is multiplied by the embedded matrix 𝑝. For example, there are 𝑛 users, the embedded dimension is 𝑚, and the embedding matrix size is 𝑚 × 𝑛. Lines refer to the user’s embedded vectors. The output layer is used to calculate the final output of the user embedded matrix and the item embedded matrix after the neural collaborative filtering layer.

#### Generalized MF Model

MF decomposes the matrix into the product of 1 or n matrices, which can solve the shortcomings of data sparseness in the previous CFA using proximity. The neural CFA model is the Generalized Matrix Factorization (GMF) model on the left. The GMF model uses the product of vectors and outputs vectors. The GMF model can be defined as Equations (6)–(9).


(6) $$p_{u}=\left[p^{1},\ldots \ldots ,p^{k}\right]$$

(7) $$q_{i}=\left[q^{1},\ldots \ldots ,q^{k}\right]$$

(8) $$\varphi \left(p_{u}{\mathrm{,q}}_{i}\right)=p_{u}\cdot q_{i}=\left[p^{q}q^{q},p^{2}q^{2},\ldots \ldots ,p^{k}q^{k}\right]$$

(9) $$y_{ui}=\alpha_{out}\left(h\left(p_{u}\cdot q_{i}\right)\right)$$

Among the above equations, $p_{u}$ and $q_{i}$ are the input into the embedded layer to obtain potential feature vectors of users and items. The relevance between the user and the item is obtained and passes through the inner product. The final prediction result is obtained and output through the output layer. 𝛼_𝑜𝑢𝑡_ denotes the Sigmoid function. $h\left(p_{u}\cdot q_{i}\right)$ represents the overall output layer.

#### Artificial Neural Network With Forwarding Structure

Multi-Layer Perceptron (MLP) is a frequently used artificial neural network with a forward structure. Each layer is connected by full connection, and the data transmission method between layers uses forward propagation. Here, MLP was used to compute each output. Back Propagation algorithm was used to find the best parameters.

The 𝑎_ℎ_ and 𝑎_𝑜𝑢𝑡_ functions are obtained from the hidden layer according to Equations (10) and (11), where (𝑎, 𝑏) refers to the input data of the input layer, 𝑤_1_ represents the weight of the hidden layer, and 𝑤_2_ denotes the weight of the output layer.


(10) $$\alpha_{h}=f_{1}\left(w_{1}\cdot \alpha \right)$$

(11) $$\alpha_{out}=f_{2}\left(w_{2}\cdot \alpha_{h}\right)$$

**Figure 5:**
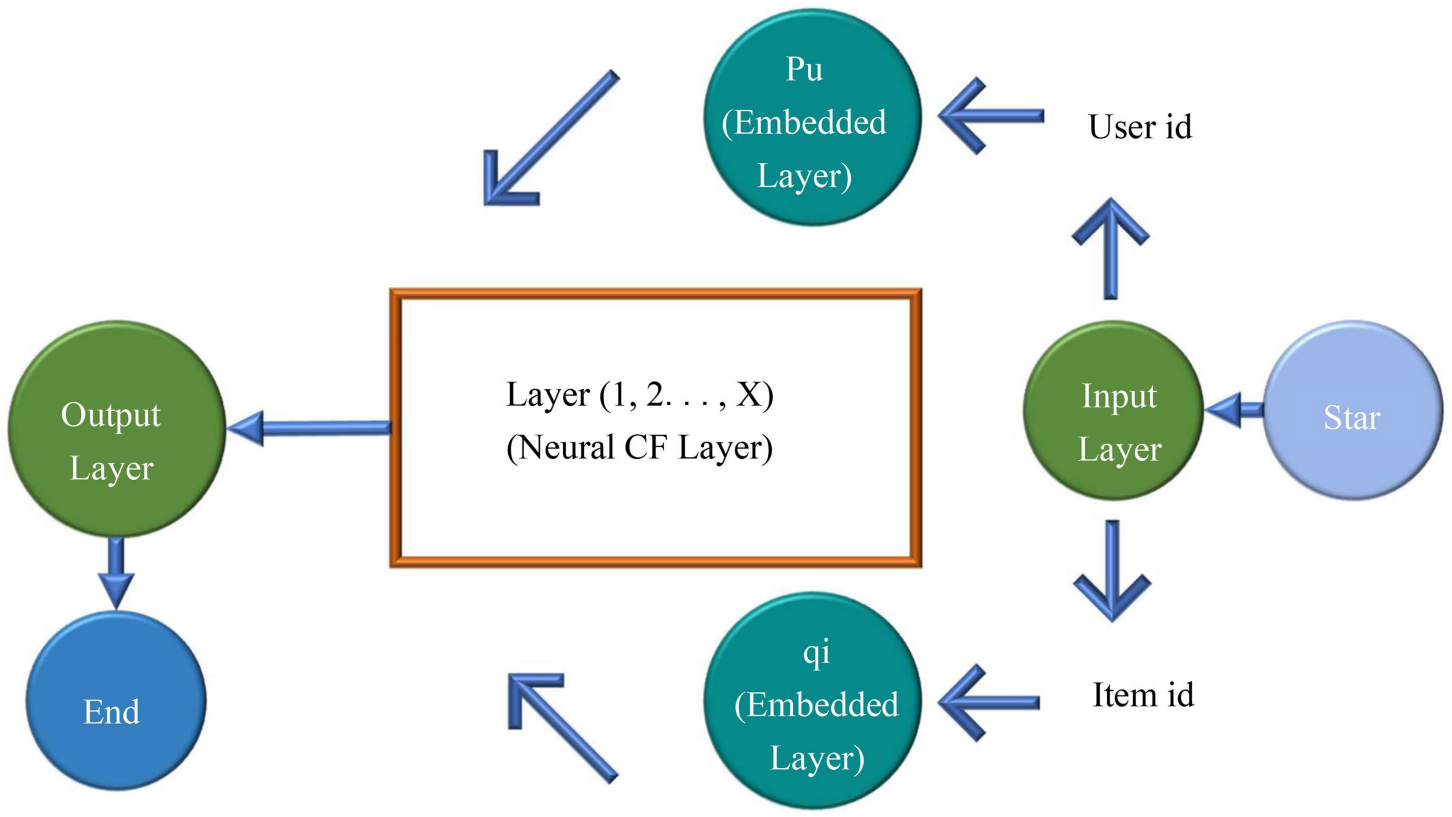
Framework of multi-layer perceptron (MLP).

The MLP framework with the special network structure can quickly and accurately find deep connections between users and objects. Figure 5 reveals the framework of MLP.

Unlike the GMF model’s processing of user and item embedded vectors in the embedding layer, MLP inputs the user and item feature vectors Pu and Qi into a multi-layer neural network to obtain the final scoring result.

### Construction of the Neural CFA Model Using Temporal Auxiliary Information

In practice, the time variable of curriculum resources has a greater effect than other resources. As time changes, the existing recommendation algorithms usually does not consider the temporal information elements. The algorithm generally defaults to the user’s preference being fixed. In the real teaching process, the user’s preference will change over time. Therefore, incorporating DL into the algorithm is still not enough, so relevant temporal auxiliary information to is added to discover the user’s dynamic preferences for the recommendation effect optimization. Compared with other recommendation models, the course resource recommendation model designed here considers time, the continuous advancement of learning stages, and the dynamic update of course resources. Each user is likely to face different learning tasks at different stages. Courses usually span a long time, and it is not essential to recommend courses that students do not see often. The K-means clustering algorithm is classified by temporal information and combined into the MLP model and the GMF model as a time feature vector to construct a neural CFA model integrating temporal auxiliary information. This model realizes a dynamic recommendation with high accuracy.

The K-means clustering algorithm is a partitional clustering algorithm. It randomly selects several numbers on the data set as the initial center value. Then compare the existing numbers in the data set with the obtained center value. The existing numbers in the dataset are then compared with the resulting central value to calculate the distance between the central value and each number. Calculations document the correlation of these numbers to the center. Each number is assigned to the cluster center closest to it. The gap between the cluster centers represents each number’s cluster, which will later be assigned a number. The algorithm loops when the cluster centers change. When the center value of the cluster does not change, the algorithm ends. This method divides the data into several categories. Equation (12) describes the distance *y*.


(12) $$y=\min\overset{n}{\underset{i=1}{\sum }}\underset{j=1,2,..k}{\min}x_{i}-\mu^{2}$$

In Equation (12), 𝑥_𝑖_ denotes the data in the data set; 𝜇 stands for the cluster center value; *k* signifies the number of initial clusters.

**Figure 6:**
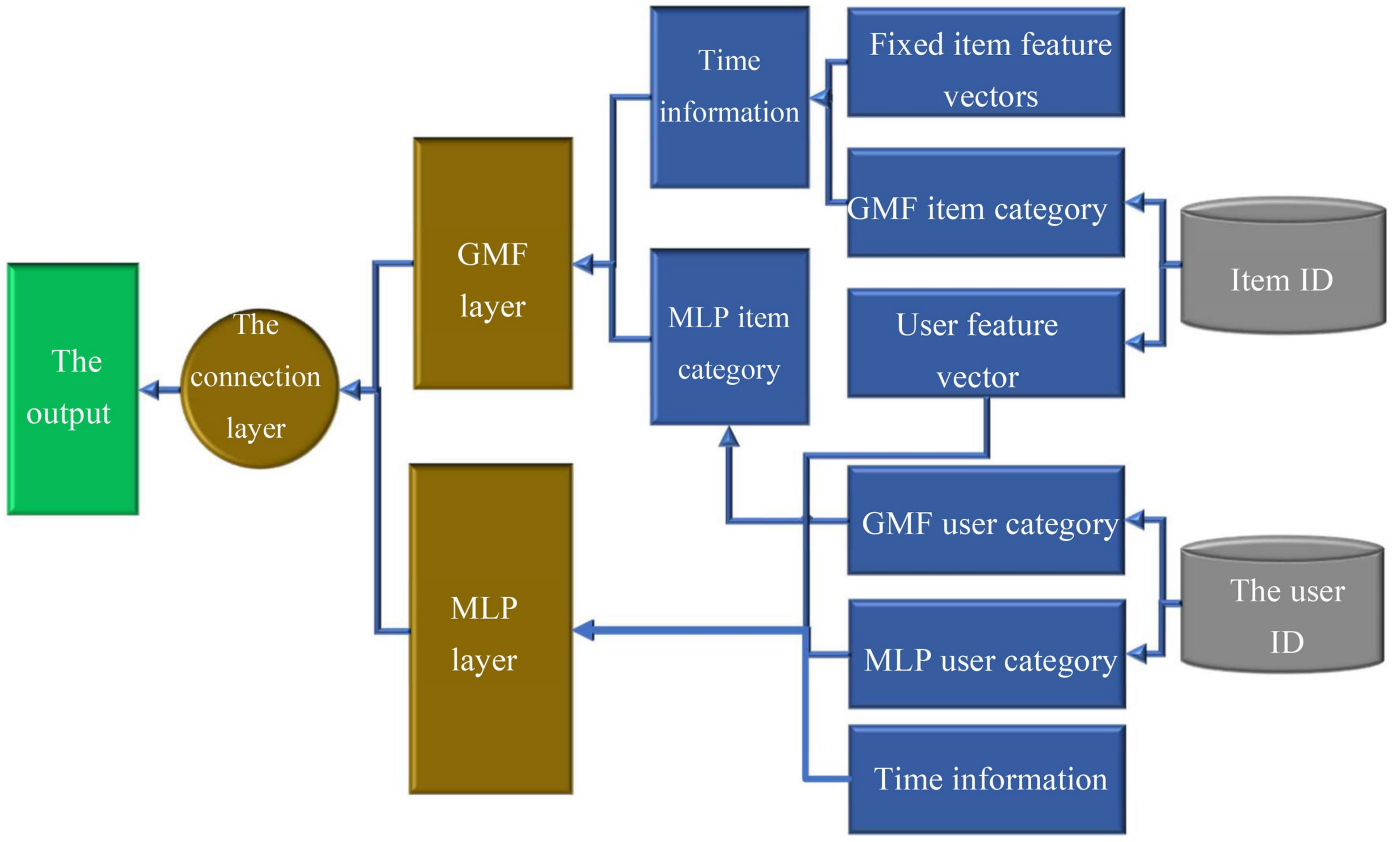
Improved Neu MF model.

K-means algorithm can find the correlation between data in the messy data information for classification. Time is unique auxiliary information. It can be infinitely large. Therefore, using time as auxiliary information in the model increases the complexity of the model. The K-means algorithm can solve this problem by mapping temporal information to an interval. The Improved Neural Matrix Factorization (Neu MF) is input as auxiliary information. Figure 6 provides the basic framework of the Improved Neu MF model.

The network layers will increase when the Improved Neu MF model conducts learning and training, slowing down the convergence during training. Batch standardization uses standard methods to apply the distribution of the input values of each layer of the neural network to accelerate the entire training speed. The batch normalization layer is used in the MLP model to alleviate the over-fitting problem when the training speed increases. The obtained deep-level feature vectors are output using the Sigmoid function after linear learning and nonlinear learning. Equations (13–17) indicate the Improved Neu MF model.


(13) $$X^{GMF}=p_{u}^{GMF}\cdot q_{i}^{GMF}+p_{i}^{GMF}\cdot t_{ui}^{GMF}$$

(14) $$X^{\mathrm{MLP}}=\alpha_{L}\left(W_{L}^{T}\left(\alpha_{L‐1}\left(\ldots \alpha_{2}\left(W_{2}^{T}\left[\begin{array}{c} p_{u}^{M}\\ q_{i}^{M}\\ t_{ui}^{M} \end{array}\right]+b_{2}\right)\ldots \right)\right)+b_{L}\right)$$

(15) $$y_{ui}=\sigma \left(h^{T}\left[\begin{array}{c} X^{GMF}\\ X^{MLP} \end{array}\right]\right)$$

(16) $$L=\frac{1}{n}\overset{n}{\underset{i=1}{\sum }}{\left|y_{ui}-y_{\mathrm{real}}\right|}^{2}$$

(17) $$L=\underset{\left(\mathrm{u,i}\right)\in D\cup D^{-}}{\sum }\left[y_{ui}\log y_{\mathrm{real}}-\left(1-y_{ui}\right)\log\left(1-y_{\mathrm{real}}\right)\right]$$

In the above equations, $p_{u}^{M}$ and $p_{u}^{GMF}$ represent the user’s input in MLP and GMF; $t_{ui}^{GMF}$ and $t_{ui}^{M}$ are the categorization information of watch time of user 𝑢 and item *i* in GMF and MLP. They are input as one-dimensional vectors.

### Data Acquisition and Experimental Validation

There are many classroom platforms online, such as NetEase Classroom and Tencent Classroom. The Scrapy framework in Python was employed to crawl the class data in the Netease classroom, generating 283,455 sets of data. The data of users with more than 12 course records was randomly selected from the data sets. Among them, the data of 5,598 users and 533 courses in the data set was used as the experimental data. Data from watched courses were used as the test set, the 100 courses that users did not watch and the rest are used as the training set. The improved CFA was compared with conventional CFA, Neu Matrix Factorization (NeuMF), and improved NeuMF algorithms from the perspectives of Root Mean Square Error (RMSE) and Mean Absolute Error (MAE) for performance verification. The learning rate of the DL model was set to 0.001, the regularization parameter was set to 5.0, the Batch Size was 128, the activation function was Relu, and the number of iterations was 5,000.

## Experimental Design and Research Results

### Experiment Design and Research Process

This study uses two metrics to measure the quality of course recommendation: MAE and RMSE. These two evaluation indicators are widely used to evaluate the pros and cons of the recommended course algorithm. Specifically, this study treats all course selection records as a positive sample. The most recent courses taken by each user are used for testing, and the remaining courses taken are used for training. K courses are randomly selected from the courses that the user has not taken as negative samples and added to the training set, so the ratio of positive and negative samples in the training set is 1:K. Here, the hyperparameter K is set to 4. In addition, it takes a lot of time to recommend and sort all courses for each user due to the large number of courses in the MOOC platform. Therefore, this paper randomly selects some courses from the ones that the target users have not taken as negative samples and adds them to the test set. In this paper, each positive sample in the test set corresponds to 19 negative samples, so each user in the test set corresponds to 20 interactions. This method is also extensively used in other related studies.

The performance of the algorithm is significantly improved when the size of the embedding layer is increased from 8 to 16. The reason is that part of the feature information is lost when the embedding layer is too small, limiting the representation ability of the model. However, the performance improvement becomes smaller when the size of the embedding layer increases from 16 to 32. This is because most of the feature information can be effectively represented at this time; continuing to increase the embedding layer has a limited improvement in the performance of the model, and at the same time, it will increase the parameters that need to be trained, thereby increasing the convergence time when training the model. The interaction between different indicators needs to be considered when evaluating the course quality. The specific research process of establishing the course quality evaluation system is as follows.

First, a hierarchical structure model is built through AHP software.

Second, the weight coefficient matrix between different indexes of course quality evaluation is established according to the index weight results.

Third, the evaluators are selected to evaluate the teaching quality according to the evaluation data of the relative importance of the indicators in the indicator evaluation system.

### Recommendation Algorithm Prediction Accuracy Test

Table 1 lists the test results of the prediction accuracy of the recommendation algorithm.

**Table 1:** Comparison between RMSE and MAE.

Index	Neu MF	CFA	Improved Neu MF
RMSE	1.372	3.362	1.251
MAE	0.825	2.953	0.625

**Figure 7:**
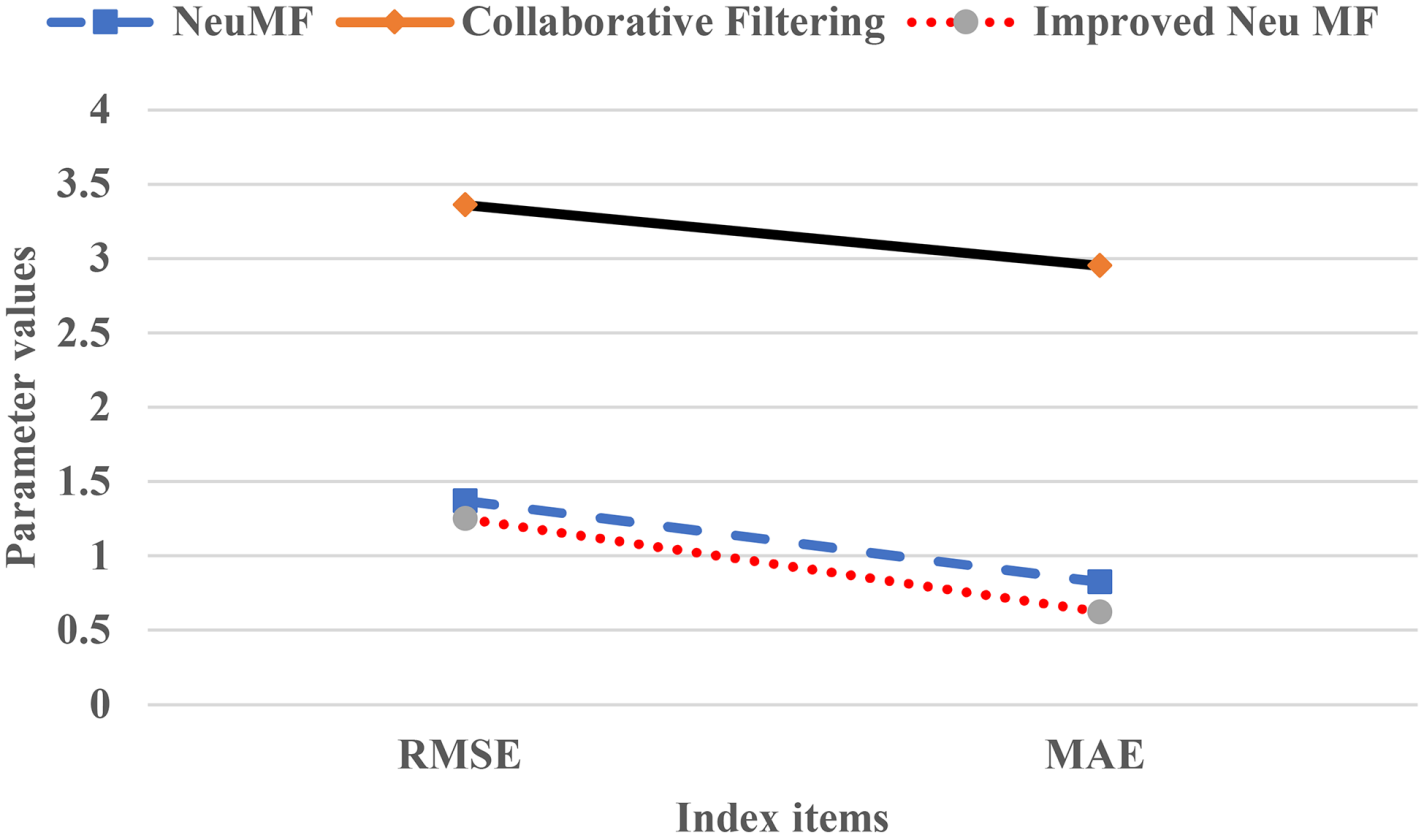
Comparison of root mean square error (RMSE) and mean absolute error (MAE).

In Table 1, the RMSE of Improved Neu MF is 1.251, and the MAE is 0.625. Compared with the other two algorithms, this algorithm has smaller errors and higher accuracy. Figure 7 presents the line chart for performance comparison with highlight the superiority of the algorithm reported here.

In Figure 7, the RMSE and MAE of the Improved Neu MF model are lower than those of Neu MF and CFA, showing an apparent advantage in the prediction precision.

### Recommendation Performance Test

Hits Ratio (HR) can precisely measure the recommended accuracy, and Normalize Discounted Cumulative Gain (NDCG) can clearly reflect the order of recommended items. The three algorithms are compared experimentally, and the results are summarized in Table 2.

**Table 2:** NDCG and HR experimental results.

	NDCG	HR
Neu MF	0.32	0.37
CFA	0.06	0.11
Improved Neu MF	0.42	0.51

**Figure 8:**
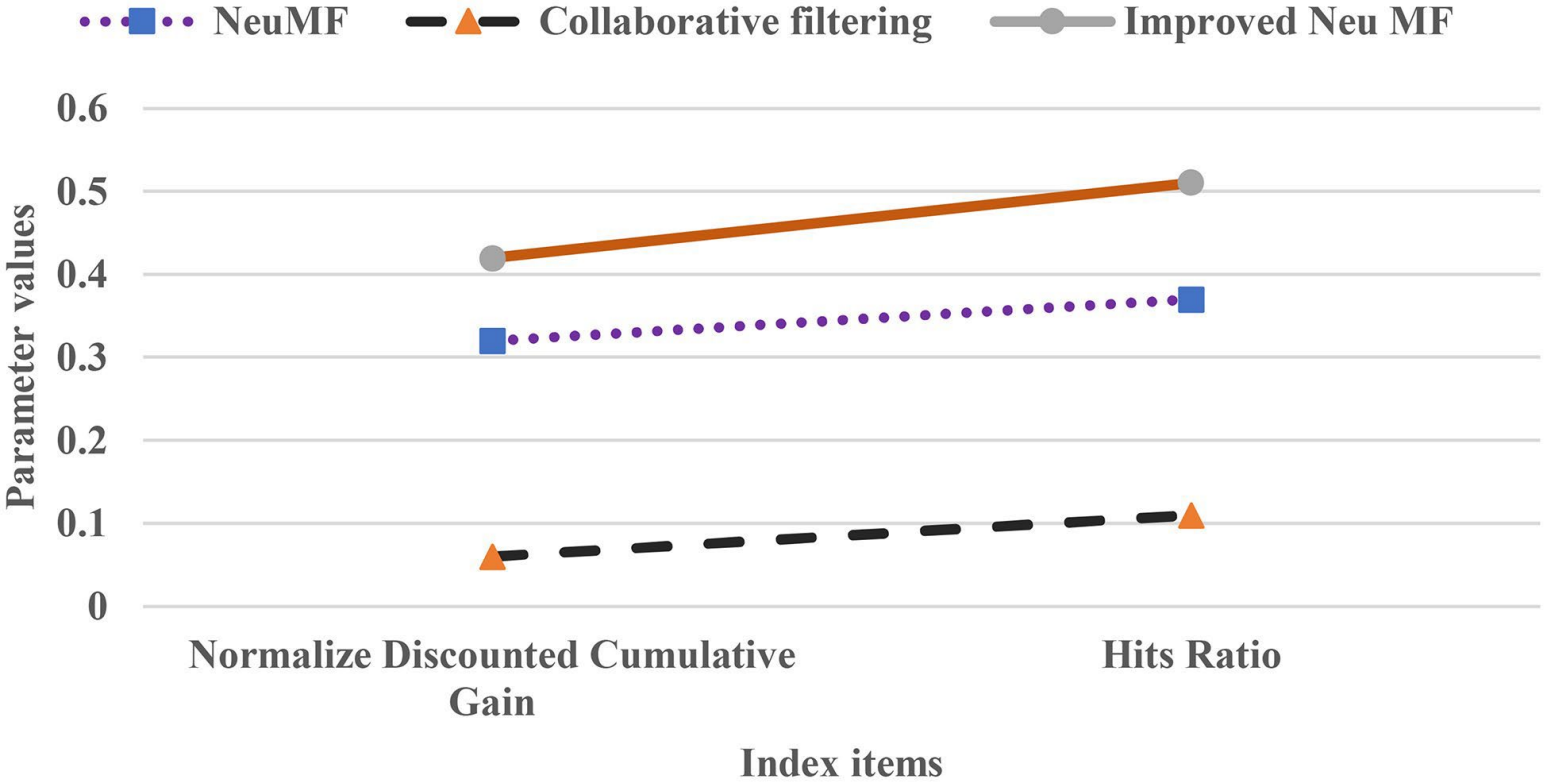
Comparison of the gap between and normalize discounted cumulative gain (NDCG) and HR.

According to Table 2, Improved Neu MF’s NDCG reaches 0.42, and HR is 0.51. Adding time information can improve the course recommendation results compared with the other two algorithms. Figure 8 is a graph to clear reflect the performance gap in terms of NDCG and HR.

In Figure 8, the NDCG and HR of the Improved Neu MF model are higher than those of the comparison algorithms. As Figure 8 and Table 2 show, the improved Neu MF algorithm has the highest evaluation index values of NDCG and HR, which are 0.36 and 0.40 units higher than CFA with the worst results, respectively, showing apparent advantages. Therefore, the performance of the algorithm is improved due to the addition of temporal information.

### Discussion

With the rapid development of the Internet economy, the complex information on the society and the network has caused a significant impact on the life of students. From the perspective of educational psychology, this research analyzes the shortcomings of the existing course recommendation algorithms and finds the unsolved problems, such as data sparsity, time information fusion and accuracy. Therefore, the deep learning algorithms, MLP and CFA algorithms, are adopted to construct an improved CFA recommendation algorithm. Training data sets and test data sets are used to verify the model results. The results demonstrate that the RMSE and MAE of the improved Neu MF model are lower than those of Neu MF and CFA, showing obvious advantages in prediction accuracy. The RMSE value of this scheme is 1.251, and the MAE value is 0.625, which are better than similar algorithms, indicating that the model has better performance after optimization and can recommend suitable courses. In addition, the improved Neu MF algorithm has the highest NDCG and HR evaluation index values, which are 0.36 and 0.40 units higher than the CFA with the worst result, respectively, with obvious advantages. Gulzar and Leema (2018) studied the curriculum recommendation system based on teaching classification method and described how scholars choose courses in various fields to meet research objectives in non-formal education. The research results showed that the course recommendation algorithm has practical reference value for data availability and course performance. Shahbazi and Byun (2022) studied an agent-based network learning system for course recommendation. They found that users face various challenges on online platforms, one of which is the identification of the real information of search results based on these resources. Therefore, this paper can provide theoretical support for the development of deep learning algorithms and has practical guiding significance for the improvement of social and political teaching strategies from the perspective of educational psychology.

## Conclusion

In the context of the rapid development of the Internet, students’ minds are easily influenced by complex information. Currently, DL-based recommendation algorithms are often used in the education field. The performance of the recommendation algorithm has a significant impact on the effect of teacher-student interaction. This work used DL to solve the problem of data sparse. Furthermore, neural CFA was designed by combining the advantages of DL and conventional CFA. Traditional MF is used to find linear relationships between users and items, and the multi-layer neural network was used to detect nonlinear relationships between users and items. This method can reduce the interference of missing data values and enhance recommendation precision. With the advancement of the teaching process, curriculum resources and teaching methods should also be changed accordingly. Therefore, the K-means clustering algorithm was applied to neural CFA through temporal data to maximize the optimization of course resources. Finally, appropriate evaluation metrics were selected to conduct simulation experiments on relevant data sets. The experimental results indicated that the improved Neural CFA model combining the advantages of DL and traditional CFA has superior performance. Teachers can analyze the absent social and political courses and students’ learning psychology based on educational psychology, promoting the teaching interaction between teachers and students. The main deficiency of the research is that there may be a certain impact on the reliability of the experimental results since this experiment only used the data of a single learning platform. Future research will combine user learning data on multiple platforms to check and optimize the model to improve the Effect of teaching strategies.

## Data Availability Statement

The raw data supporting the conclusions of this article will be made available by the authors, without undue reservation.

## Ethics Statement

The studies involving human participants were reviewed and approved by Chengdu Normal University Ethics Committee. The patients/participants provided their written informed consent to participate in this study. Written informed consent was obtained from the individual(s) for the publication of any potentially identifiable images or data included in this article.

## Author Contributions

ZC: writing-original draft preparation and methodology. LW: conceptualization, software, and validation. XH: data curation and formal analysis. PC: writing—review and editing and visualization. HW: writing-original draft preparation. All authors listed have made a substantial, direct, and intellectual contribution to the work and approved it for publication.

## Conflict of Interest

The authors declare that the research was conducted in the absence of any commercial or financial relationships that could be construed as a potential conflict of interest.

## Publisher’s Note

All claims expressed in this article are solely those of the authors and do not necessarily represent those of their affiliated organizations, or those of the publisher, the editors and the reviewers. Any product that may be evaluated in this article, or claim that may be made by its manufacturer, is not guaranteed or endorsed by the publisher.
